# Expression of an epidermal growth factor-transdermal peptide fusion protein in *Arabidopsis thaliana* and its therapeutic effects on skin barrier repair

**DOI:** 10.3389/fpls.2025.1573193

**Published:** 2025-04-04

**Authors:** Guangdong Yu, Shisheng Lin, Xulong Huang, Shuang Gao, Chengyang Song, Farid Khalilov, Qiongzhen Chen, Nipatha Issaro, Jiali Xiao, Xiashun Xu, Junchao Wang, Wengang Zhao, Yunpeng Wang, Nuo Xu

**Affiliations:** ^1^ College of Life and Environmental Sciences, Wenzhou University, Wenzhou, China; ^2^ Institute of Agricultural Biotechnology, Jilin Academy of Agricultural Sciences (Northeast Agricultural Research Center of China), Changchun, China; ^3^ Technology Development Department, Zhejiang Tianqu Beiben Instrument Technology Co., Ltd, Wenzhou, China; ^4^ Institute of Agricultural Biotechnology, Jilin Academy of Agricultural Sciences (Northeast Agricultural Research Center of China), Chonburi, Thailand; ^5^ Technology Development Department, Zhejiang Tianqu Beiben Instrument Technology Co., Ltd., Wenzhou, China

**Keywords:** epidermal growth factor, transdermal peptide, fusion protein, a. thaliana expression system, skin barrier damage repair

## Abstract

Epidermal growth factor (EGF) is recognized for its role in regulating keratinocyte proliferation and differentiation, thereby facilitating the restoration of impaired skin barriers. Nevertheless, challenges related to the penetration and safety of EGF remain to be resolved. In this study, we evaluated the efficacy of TDP1, a transdermal peptide, in enhancing the penetration of EGF through murine skin, utilizing EGF expressed in *A. thaliana*. The coding sequences of the TDP1 and EGF genes were cloned as a fusion construct into a plant expression vector. The resulting plasmid, pGM3301-TDP1-EGF, was introduced into *A. thaliana* via the floral dip method. Positive clones were identified using polymerase chain reaction (PCR). High-expression strains were selected through Western-blot analysis and enzyme-linked immunosorbent assay (ELISA). Homozygotes plants were obtained through self-pollination. The impact of the TDP1-EGF fusion protein on the restoration of a compromised epidermal barrier was assessed using dermatoscopy. Keratinocyte (KC) proliferation was examined via hematoxylin and eosin (H&E) staining, while KC differentiation, lipid synthesis, and inflammatory factors were analyzed using reverse transcription quantitative PCR (RT-qPCR) and immunohistochemistry. Compared to other expression systems, the *A. thaliana* system utilized for TDP1-EGF expression offers the advantages of being devoid of toxicity from endogenous plant substances, rendering it both safe and suitable for scalable production of the recombinant protein. The yield of the TDP1-EGF fusion protein expressed in *A. thaliana* accounted for 0.0166% of the total soluble protein. EGF conjugated with TDP1 displayed enhanced transdermal activity compared to unconjugated EGF, as evidenced by the Franz diffusion cell assay. Furthermore, the biological efficacy of the TDP1-EGF fusion protein surpassed that of EGF alone in ameliorating epidermal barrier damage in a murine skin injury model. This research has the potential to revolutionize the development and delivery of skincare products and establishes a foundation for the application of molecular farming in skin health.

## 1 Introduction

The skin barrier is a regular structure composed of the stratum corneum and intercellular lipids in a “brick and mortar” arrangement, and its main function is to protect the skin against mechanical, physical, chemical, and biological external stimuli and maintain the stability of the internal environment of the body (Montero-Vilchez et al., 2021; Panzuti et al., 2020). It is the first line of defense against external factors that may harm the body (Xu et al., 2019; Goverman, 2014). The skin barrier is formed by the basal layer keratinocytes proliferating and differentiating in an orderly manner as they migrate upward to form the spinous layer, granular layer, and eventually the stratum corneum (Segre, 2006; Joly-Tonetti et al., 2021). A healthy skin barrier is one that undergoes a continuous process of self-renewal (Labarrade et al., 2015; Hänel et al., 2013; Candi et al., 2005; Lim, 2021). When the structure of the skin barrier is damaged, harmful substances and pathogens from the external environment can invade the skin and disrupt its internal homeostasis (Yosipovitch et al., 2019). This will then hinder the differentiation of keratinocytes, leading to abnormal proliferation and an inability to form a healthy skin barrier, eventually giving rise to a series of skin problems (Wikramanayake et al., 2014; Lee et al., 2006). Thus, regulating keratinocyte proliferation and differentiation is essential for preserving skin barrier integrity and safeguarding skin health.

Epidermal growth factor (EGF) is the first discovered growth factor, and it is a 53-amino acid peptide that plays an important role in regulating cell growth, proliferation, and differentiation (Liou et al., 1997). EGF promotes the phosphorylation of AKT/PI3K, thereby activating the AKT/PI3K signaling pathway. The AKT/PI3K signaling pathway represents a critical intracellular signaling network that plays a pivotal role in regulating diverse physiological processes, including cell metabolism, proliferation, survival, and migration, eventually resulting in elevated expression of differentiation markers (e.g., filaggrin) in keratinocytes (KC) (Oda et al., 2005; Haase et al., 2003). Thus, its function is also to promote the differentiation of keratinocytes, and such a function can facilitate the repair of damaged epidermal barriers (Zhang et al., 2014; Chen et al., 2022b; Li et al., 2021). However, the transdermal efficacy of EGF is rather low because the large size of EGF (6,045 Da) makes it difficult for the peptide to effectively penetrate the membrane of KC (Adamson and Rees, 1981). The transdermal peptide (TDP1), a short peptide consisting of 11 polar amino acids, served as a carrier to facilitate the transdermal delivery of protein therapeutics to the dermal layer. Studies have shown that simply mixing transdermal peptide with insulin and applying it to the skin of diabetic rats can effectively lower their blood glucose levels (Chen et al., 2006). A study demonstrated that fusing TDP1 with EGF enhances the efficacy of EGF in repairing compromised skin barriers.

TDP1 is derived from TD1, a bacteriophage-derived transdermal peptide, through codon optimization for plant-based expression. Plant bioreactors offer the advantage of performing post-translational modifications on eukaryotic proteins, a capability absent in prokaryotic expression systems (Webster and Thomas, 2012). Expressing the TDP1-EGF fusion protein would ensure that the structure and function are very close to those of the natural protein, thus guaranteeing the efficacy of both TDP1 and EGF (Huang and McDonald, 2012). Plant bioreactors do not contain pyrogens, endotoxins, or other allergenic substances, thus reducing the potential risk of allergenicity and pathogenicity that may contaminate the exogenously expressed proteins (Moustafa et al., 2016; Ren et al., 2018). The plant bioreactor system can stably express exogenous proteins with lower costs, making it easier to achieve industrial application (Ma et al., 2003). Although the expression level of foreign proteins in *A. thaliana* is below 0.1% of total protein—a concentration necessary for commercial applications—the use of a whole-plant expression strategy renders this approach viable. This approach not only can boost the bioaccumulation of the foreign proteins but also ensure stable inheritance and significantly reduce labor costs (Zhang et al., 2021). However, some plant expression systems are not suitable for expressing skin care products; for example, studies have shown that nicotine can affect protein composition and damage organelles, particularly disrupting mitochondrial and peroxisomal reactive oxygen species (ROS) homeostasis in the 3D human skin model EpiDerm (Pozuelos et al., 2022). These alterations may aggravate skin infections, impede wound healing, and promote oxidative damage in skin cells. Consequently, due to safety concerns, *Nicotiana tabacum* is deemed unsuitable for producing skin care products. In contrast, more suitable plant candidates for such applications include *Arabidopsis thaliana*, legumes, fruits, and vegetables. As a model plant, *A. thaliana* possesses several advantageous traits compared to other species, including high transformation efficiency, robust adaptability, rapid growth, a short life cycle, and substantial biomass (Vasseur et al., 2018; Koenig and Weigel, 2015). Furthermore, *A. thaliana* is distinguished by its elevated anthocyanin content (Kovinich et al., 2014), which may synergistically augment the skin care benefits of the TDP1-EGF fusion protein. Moreover, Arabidopsis extract is officially recognized and registered as a cosmetic raw material in the International Nomenclature of Cosmetic Ingredients (INCI) catalog. The expressing TDP1-EGF in *A. thaliana* offers several advantages over its expression in bacterial system. Foremost among these is the enhanced safety of the final product, a critical consideration given its intended future application in humans. Additionally, plant-based expression eliminates the need for extensive protein purification, as the target protein can be utilized as a crude extract of total protein, thereby simplifying the process and substantially reducing costs associated with protein drug preparation (Michalak, 2023). However, the efficacy of using a crude protein extract—subjected only to centrifugation to isolate the soluble fraction—depends on achieving a sufficiently high expression level of the target protein. In this study, TDP1 and EGF were co-expressed as a fusion protein to enhance the transdermal delivery of EGF. The coding sequence of this fusion gene was optimized for expression in *A. thaliana* to improve safety and preserve the functionality of the fusion protein. Subsequently, A murine model of skin barrier impairment was employed to assess the safety and efficacy of the fusion protein. The primary goal was to establish to a feasible strategy for repairing skin barrier damage and treating associated skin disorders while evaluating the practicality of plant-based expression systems for fusion protein production. These findings offer valuable insights into the therapeutic potential of EGF in skin health and provide a foundation for investigating the mechanisms underlying fusion protein-mediated skin barriers repair and for developing novel skincare raw materials.

## 2 Materials and methods

### 2.1 Construction plant expression vector

The TDP1 gene was amplified from the plasmid pET-15b-TDP1 via PCR using gene-specific primers: 5’-ATGCCCATGGGTCATATGGCGTGCAG-3’ (forward) and 5’-ATGGGGTACCACCGCCACCGCC-3’ (reverse). Similarly, the EGF gene was amplified from pET-15b-EGF using primers 5’-ATGGGGTACCAACAGCGACT-3’ (forward) and 5’-ATCGGGTAACCCAGGATCCTTAGC-3’ (reverse). The resulting PCR products were cloned into the pEASY^®^-T1 vector as a fusion construct, generating pEASY-TDP1-EGF, which was subsequently transformed into *Escherichia coli*. Positive clones were identified through PCR screening and confirmed by DNA sequencing. The pEASY-TDP1-EGF plasmid was extracted from a verified clone, digested with *NcoI* and *BstEII*, and the TDP1-EGF DNA fragment was ligated into the *NcoI*/*BstEII*-digested plant expression vector pGM3301, yielding pGM3301-TDP1-EGF. Cleavage sites are detailed in **Supplementary Table S1**. The ligation product was transformed into Trans-T1 *E. coli*, and positive clones were selected via PCR and DNA sequencing. The plasmid isolated from one positive clone was then introduced into *Agrobacterium tumefaciens* strain EHA105. The recombinant *A. tumefaciens* EHA105 was subsequently used to transform *A. thaliana* for TDP1-EGF expression.

### 2.2 Transformation of arabidopsis strains and screening of transgenic plants

YEP medium was utilized to culture *Agrobacterium* until an OD600 of 0.8-1.0 was achieved, after which the cells were harvested by centrifugation at 4,000 × g/for 20 minutes at 4°C. The cells were resuspended in transformation solution (50 g/L of sucrose, 2.2g/L of ½ MS, 200 μL/L of surfactant, pH 5.8) and then used to infect *A. thaliana*, performed according to a previous study (Harrison et al., 2006; Purwantoro et al., 2023). After infection, the plants were incubated darkness for 48 hours, then cultivated under a 16-hour light/8-hour dark photoperiod with a light intensity of 50-100 µmol m^-2^ s^-1^. Seeds from the T1 generation were harvested, and positive clones were initially screened for growth on solid MS medium (Murashige and Skoog medium) supplemented with 11 mg/mL glufosinate. MS medium, a foundational formulation, is widely utilized in plant tissue culture. In this study, solid MS medium was utilized primarily for the germination and development of *Arabidopsis thaliana* seeds. To screen for transgenic *A. thaliana* seedlings, the medium was supplemented with glufosinate (1 L MS formulation: 2.2 g MS salt mix, 10 g sucrose, PH 5.7, 6.5 g plant agar). Plants exhibiting normal growth were transplanted into individual soil-filled pots, with one plant per pot, and seeds were harvested from each plants to obtain the T2 generation. Based on the T2 segregation ratio, heterozygous plants carrying a single copy of the recombinant gene were selected to generate the T3 generation, yielding homozygous plants for the recombinant gene.

### 2.3 RT-qPCR analysis of transgene expression in transgenic arabidopsis

To confirm the integration of the TDP1-EGF fusion gene into the *A. thaliana* genome, transgenic plants from the T3 generation were initially screened via PCR. Genomic DNA was extracted from whole plant and subjected to PCR analysis using gene-specific primers: 5’-AACAGCGACTCTGAATGCCCGC-3’ (forward) and 5’-GCGCAGTTCCCACCACTTCAGGTC-3’ (reverse). Following verification, seeds from these plants were germinated to produce additional plants for gene expression analysis. Total RNA was isolated from distinct tissues (roots, stems, leaves, and flowers), and RNA concentrations was quantified. For each sample, 1 μg of total RNA was reverse transcribed using the PrimeScript RT reagent Kit (Takara, Dalian, China). Quantitative real-time PCR (qPCR) was conducted on a LightCycler 96 system (Roche, Basel, Switzerland) with SYBR Green Master Mix (Applied Biosystems, Foster City, CA, USA) and gene-specific primers: 5’-ATGGCGTGCAGCAGTAGCCCGAG-3’ (forward) and 5’-AACAGCGACTCTGAATGCCCGC-3’ (reverse). Total RNA was extracted from various *A. thaliana* tissues (roots, stems, leaves, and flowers) using the pBiozol Plant Total RNA Kit (BioFLUX-Pion Inc., USA), and TDP1-EGF mRNA expression levels in these tissues were quantified via RT-qPCR. Total protein was extracted from positive clones and analyzed by SDS-PAGE using Tricine gel. The TDP1-EGF band was detected by Western blot with an anti-EGF antibody. The yield of TDP1-EGF, expressed as a percentage of total plant protein, was determined using an ELISA kit (MEIMIAN, China, MM-0184H1), and plants exhibiting high expression levels were selected for subsequent protein production experiments.

### 2.4 Hydrogel preparation

To formulate a hydrogel for topical application, 1.2 g of carbomer, 10 g of glycerin, and 6 g of propylene glycol were combined with 20 g of water and stirred slowly to achieve a homogeneous mixture. Subsequently, 1.2 g of triethanolamine was added, and the mixture was stirred gently until a hydrogel matrix formed. The hydrogel matrix was sterilized by autoclaving at 1214°C for 15 minutes, and after cooling to room temperature, supplemented with a sterile TDP1-EGF solution to a final concentration of 35 ng/mL hydrogel. The resulting TDP1-EGF hydrogel was stored in a refrigerator at 4°C until use. A Hydrogel containing only EGF was prepared following the same procedure.

### 2.5 Transdermal absorption of the fusion protein TDP1-EGF

Six male mice were randomly assigned to three groups, with two mice per group. To assess the transdermal penetration of TDP1-EGF and EGF, the dorsal skin of two mice from each group was depilated. A 3 cm × 3 cm area of the dorsal skin was secured over the mouth of a conical flask filled with normal saline, ensuring the inner skin surface contacted the saline. Hydrogel containing either TDP1-EGF or EGF was uniformly applied to the depilated skin. The saline was continuously agitated using a magnetic stirrer, and 1 mL samples were collected hourly, stored at 4°C, with the volume replenished by adding 1 mL of normal saline. Concentrations of TDP1-EGF and EGF in each sample were quantified using an ELISA kit.

### 2.6 Animal model and treatments

To establish a murine model of skin barrier damage, the dorsal skin of mice was initially shaved with a razor. Subsequently, the hairless dorsal region was treated with Veet depilatory cream (Hubei, China) for 90 seconds and then cleaned with a pad moistened with warm, sterile water. Given that depilatory cream may induce mild skin irritation, a 24-hour interval was observed post-depilation before subjecting the skin to physical disruption through repeated adhesive tape stripping. Accordingly, 24 hours after depilation, the dorsal skin of the mice was stripped multiple times using adhesive tape (3M600 Scotch Tape, 3M Corporation, USA). The degree of barrier impairment was evaluated by measuring transepidermal water loss (TEWL), with a VapoMeter (Delfin Technologies, Finland). A TEWL value of greater than 35 mg/cm^2^/h was considered adequate damage in this model (Jing et al., 2024; Fusté et al., 2019). Subsequently, the mice were randomly allocated into five groups, with five mice per group. The control group remained untreated. The remaining groups underwent tape stripping were then treated with either hydrogel alone (vehicle group), hydrogel containing the soluble protein fraction from wild-type *Arabidopsis thaliana* (Arabidopsis group), hydrogel with purified EGF (EGF group), or hydrogel containing the soluble protein fraction from TDP1-EGF transgenic *A. thaliana* (TDP1-EGF group). The condition of the dorsal skin was assessed every 12 hours through photography, dermoscopy, and TWEL measurements.

### 2.7 Histology examination

To evaluate the efficacy of TPD1-EGF in promoting skin barrier repair, changes in epidermal thickness of the dorsal skin of mice were assessed following treatment. Initially, dorsal skin tissue was soaked in 4% paraformaldehyde for 24-48 hours and subsequently rinsed with running tap water for 4-6 hours. The tissue samples were then immersed in 70% ethanol overnight and dehydrated through a graded series of ethanol solutions (80%, 95%, and 100%), followed by 100% xylene. Subsequently, the samples were sectioned into 6 μm-thick slices, deparaffinized in xylene, and rehydrated using a descending ethanol gradient (95%, 90%, 80%, 70%, 0%). The rehydrated sections were stained with hematoxylin and eosin (HE; Solarbio, Beijing, China) and examined under a DM3000 microscope (Leica, Wetzlar, Germany) to measure epidermis thickness. To examine the impact of TPD1-EGF on skin cell differentiation, two differentiation-related proteins, involucrin and loricrin, were selected for analysis. Initially, rehydrated tissue sections were immersed in sodium citrate antigen retrieval solution (Solarbio, Beijing, China). Subsequently, the sections were treated dropwise with either a 1:500 dilution of anti-involucrin antibody (Proteintech, Wuhan, China) or a 1:200 dilution of anti-loricrin antibody (Absin, Shanghai, China), and incubated overnight at 4°C. The sections were then washed three times with PBS and incubated with a horseradish peroxidase (HRP)-conjugated secondary antibody at 37°C for 4 hours. Following this, 3,3’-diaminobenzidine (DAB) chromogen was applied, and the sections were counterstained with hematoxylin. The stained sections were then examined under a DM3000 microscope (Leica, Wetzlar, Germany) to evaluate histological changes.

#### 2.8 Immunofluorescence analysis

Following antigen retrieval with sodium citrate solutions as described in Section 3.7, tissue sections were incubated with either a 1:200 dilution of anti-PCNA antibody (Cat. No. 2586; RRID: AB_2160343; Cell Signaling Technology, Beverly, MA, USA) or a 1:200 dilution of anti-K16 antibody (Cat. No. 2586; RRID: AB_2160343; Cell Signaling Technology, Beverly, MA, USA) at 4°C overnight. The sections were then washed three times with PBS and incubated with an Alexa Flour 568-conjugated secondary antibody for 1 hour at 37°C. Subsequently, the sections were mounted using an antifade medium containing DAPI (Invitrogen, Life Technologies, Carlsbad, CA, USA) and examined using a Ti2-E & CSU-W1 confocal fluorescence microscope (Nikon, Tokyo, Japan).

### 2.9 Statistical analysis

Data analysis and statistical procedures adhered to the guidelines and requirements outlined by *Frontiers in Plant Science* for experimental design and analysis. Mice were randomly and blindly allocated to groups, with each group consisting of an equal number of mice. Technical replicates were averaged prior to statistical analysis and excluded from calculation of variance parameters. Differences between pairs of groups were evaluated using an unpaired Student’s t-test. Statistical analyses were performed using GraphPad Prism 6.01 (GraphPad Software, San Diego, CA, USA). Multicomponent data and multiple group comparisons were assessed via one-way analysis of variance (ANOVA) followed by Dunnett’s test. Statistical significance was determined at an F-value corresponding to *P* < 0.05 in the one-way ANOVA. The reported group size represents the number of independent values (n = 4) before being statistically analyzed. All quantitative data are presented as the mean ± standard error of the mean (SEM).

## 3 Results

### 3.1 Distribution of TDP1-EGF in transgenic arabidopsis thaliana

**Figure 1:**
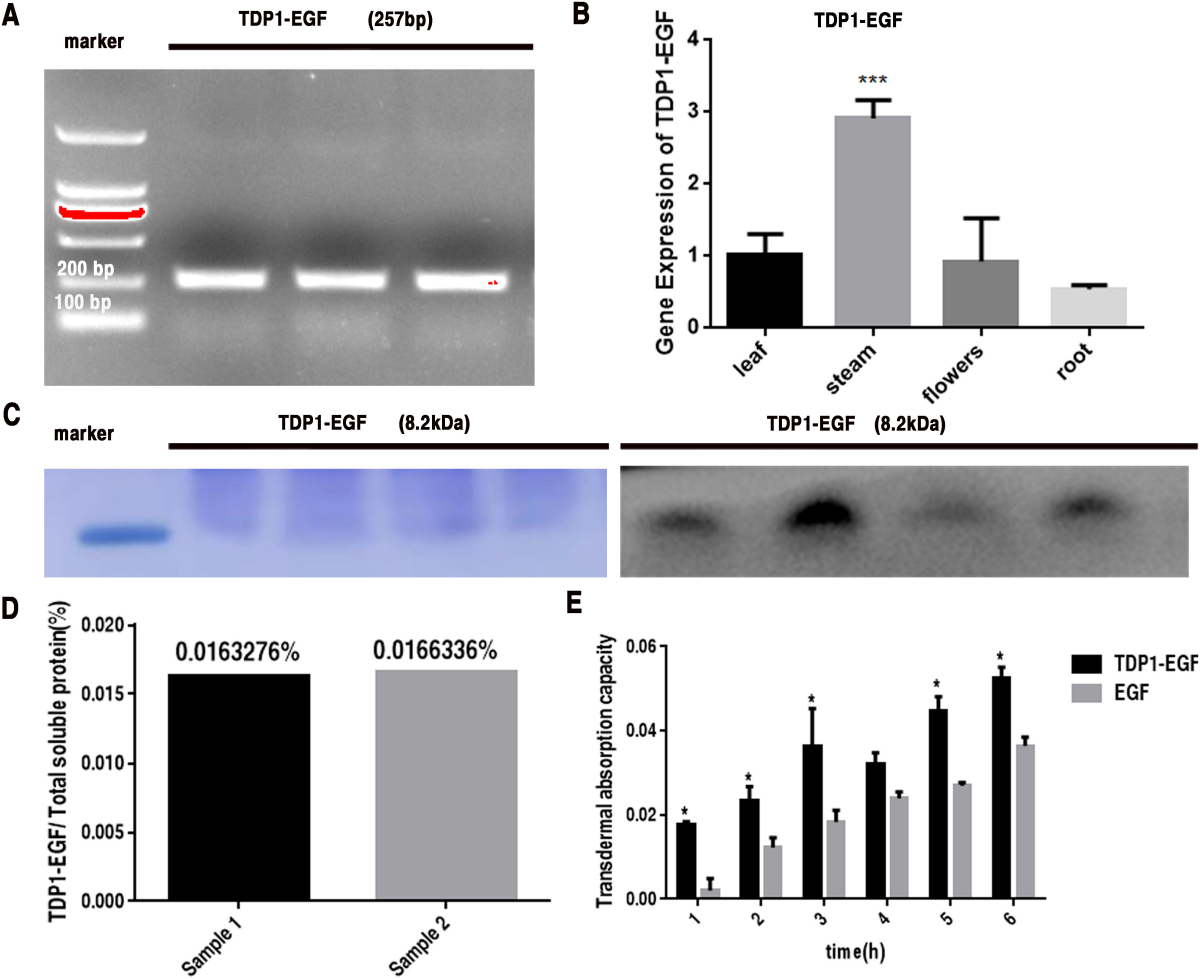
Analysis of TDP1-EGF Expression in *Arabidopsis thaliana*. **(A)** PCR detection of the TDP1-EGF DNA fragment in positive transgenic plants. **(B)** RT-qPCR analysis of TDP1-EGF transcript levels across various tissues of positive transgenic plants. **(C)** SDS-PAGE and Western blot analysis of total protein extracted from positive transgenic plants. **(D)** ELISA-based quantification of TDP1-EGF protein within the total soluble protein extracted from whole transgenic plants. **(E)** Franz diffusion cell assay assessing the transdermal activity of TDP1-EGF from total protein extracts compared to an EGF standard. All graphical data are presented as mean ± SEM from two or three independent experiments. Statistical significance is denoted by ‘*’ (*P* < 0.05) and ‘**’ (*P* < 0.01).

To assess the expression levels of the TDP1-EGF fusion gene in *A. thaliana*, genomic DNA was extracted from glufosinate-resistant plants for molecular analyses. Initial PCR screening confirmed the integration of the TDP1-EGF gene into the *A. thaliana* genome (**Figure 1A**). The expression of TDP1-EGF in the transgenic *A. thaliana* plants was further evaluated using RT-qPCR. Total RNA was extracted from the roots, leaves, stems, and flowers of transgenic plants and subjected to RT-qPCR to quantify TDP1-EGF transcript levels. Results indicated the presence of TDP1-EGF transcripts across all examined tissues, with significantly elevated levels in stems compared to other tissues (**Figure 1B**). RT-qPCR Primer sequences are provided in **Supplementary Table S2**. From the transgenic lines analyzed, four exhibiting high TDP1-EGF transcript abundance were selected for protein expression analysis. Total protein extracted from whole plants revealed a band of approximately 8.2 kDa via SDS-PAGE and Western blot analysis using an anti-EGF antibody, aligning with the predicted molecular mass of the TDP1-EGF fusion protein (**Figure 1C**). The TDP1-EGF fusion protein constituted approximately 0.016% of the total soluble protein extracted from transgenic plants (**Figure 1D**). The transdermal properties of TDP1-EGF were assessed using a Franz diffusion cell assay relative to an EGF standard, demonstrating a time-dependent increase in fusion protein accumulation in the receiver compartment, with the fusion protein consistently exhibiting significantly greater transdermal efficacy than the EGF standard across all time points (**Figure 1E**).

### 3.2 Cultivation of homozygous fusion protein arabidopsis thaliana

**Figure 2:**
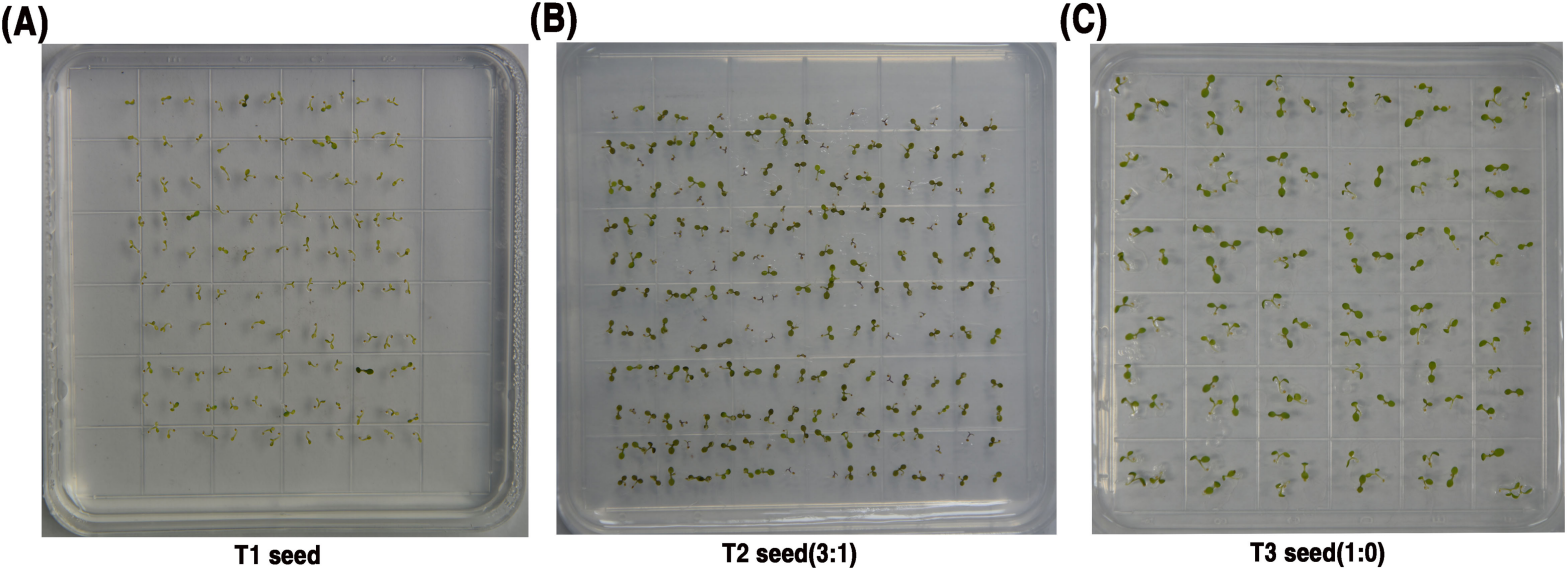
Selection of Transgenic Arabidopsis thaliana Plants Homozygous for the TDP1-EGF Gene. **(A)** Screening T1 transgenic seedlings; **(B)** Screening of T2 generation transgenic seedlings; **(C)** Screening T3 transgenic seedlings.

To establish homozygous and genetically stable positive plants, a substantial quantity of seeds was harvested. These seeds were cultivated on MS basal medium supplemented with 11 mg/L glufosinate (**Figure 2A**). Following Mendelian inheritance principles, T2 seeds were collected from individual transgenic *A. thaliana* plants (**Supplementary Figure S3B**). Heterozygote (Aa) = 3:1 was identified through screening of T2 seeds (**Figure 2B**). Subsequently, homozygote (AA) = 1:0 was selected from T3 generation seeds (**Figure 2C**).

### 3.3 TDP1-EGF enhances epidermal barrier repair

**Figure 3:**
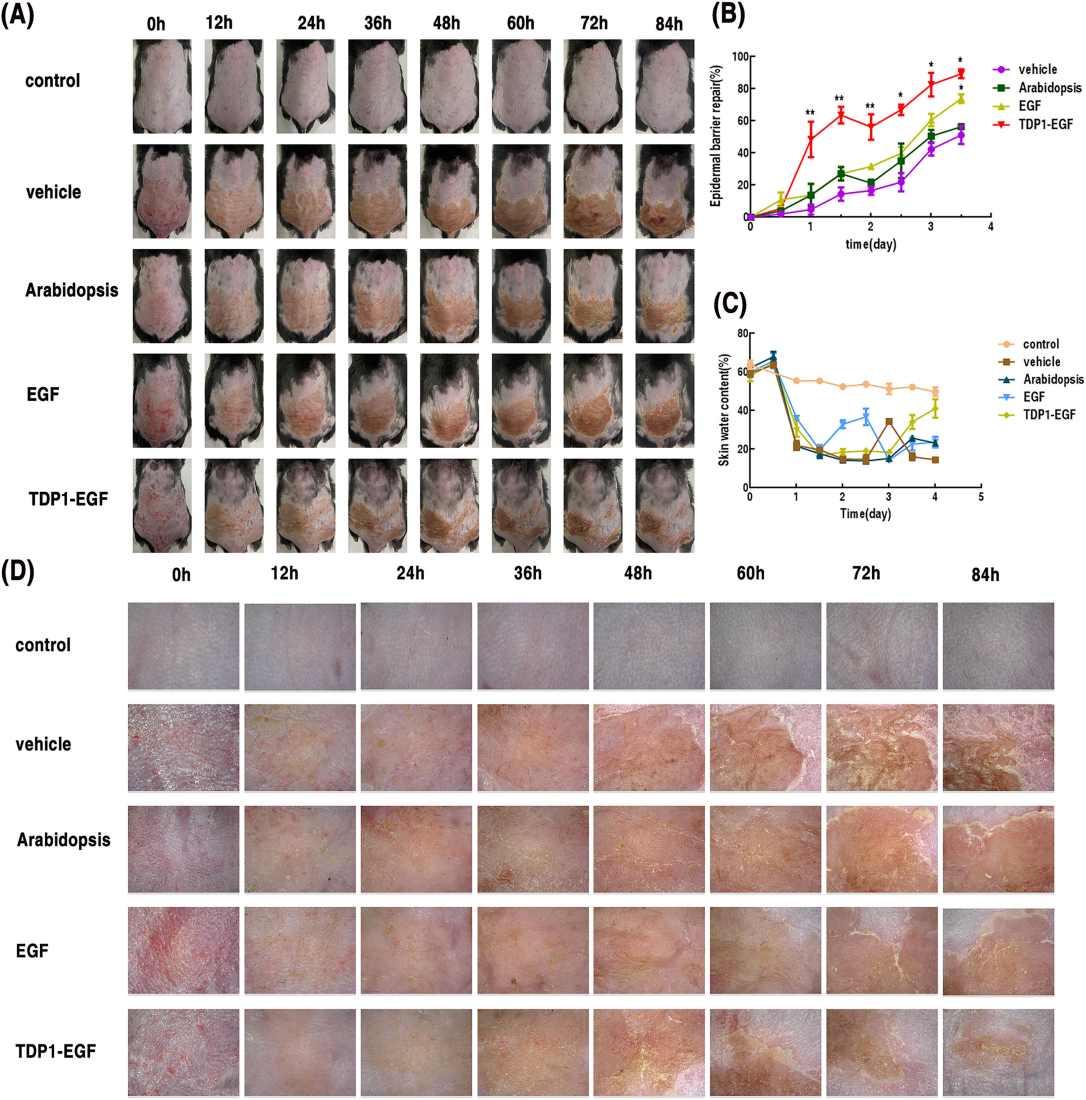
Effects of TDP1-EGF on epidermal barrier repair in mice. **(A)** Photographs of damaged dorsal skin area at various time points. **(B)** Assessment of skin barrier damage via transepidermal water loss (TEWL, mg/cm^2^/h). Barrier repair rate was calculated as: [(TEWL value after molding – TEWL value at a specific time point)/(TEWL value after molding – TEWL value before molding)] ×100%. **(C)** Skin hydration levels measured using a skin moisture analyzer. **(D)** Dermoscopic observation of the damaged murine skin barrier across multiple time points. Data are presented as mean ± SEM (n = 5). Statistical significance is denoted by ‘*’ (*P* < 0.01) and ‘**’ (*P* < 0.001).

To assess the therapeutic efficacy of TDP1-EGF in epidermal barrier recovery, a standardized murine model of skin barrier damage was established via tape stripping, utilizing transepidermal water loss (TEWL) as the primary measure of barrier function. The TDP1-EGF-treated group demonstrated superior wound healing properties compared to other experimental groups. At 24 hours post-injury, this group displayed earlier scab formation and significantly reduced scab areas. By 36 hours, distinct white marginal spots, slight bulging, and initial signs of shedding were observed in the TDP1-EGF, EGF, and *Arabidopsis* groups, in contrast to the vehicle group. Notably, while the vehicle, *Arabidopsis*, and EGF groups showed minimal changes in scab area between 36 and 60 hours, the TDP1-EGF group demonstrated progressive white edge formation and scab detachment. At 72 hours, the TDP1-EGF group exhibited a substantial reduction in scab area, with emerging stratum corneum in shed regions, though vascular network development remained incomplete. By 84 hours, unlike the delayed and less pronounced scab detachment in other groups, the TDP1-EGF group demonstrated significant scab reduction, accompanied by clear neovascularization in previously shed areas, approximating normal skin morphology (**Figure 3A**). TEWL measurements, conducted at 12-hour intervals, consistently indicated enhanced epidermal barrier recovery in the TDP1-EGF group, with notably faster recovery rates than other groups prior to 72 hours (**Figure 3B**). Skin hydration analysis at 84 hours revealed significantly elevated moisture content in the TDP1-EGF group (**Figure 3C**). Dermoscopic analysis identified early pigmented spots and hyperkeratosis in the vehicle and *Arabidopsis* groups at 24 hours. Whereas the TDP1-EGF group developed characteristic red and white spots at 36 hours, followed by progressive crust shedding and new cuticle formation at 60 and 72 hours, culminating in neovascularization by 84 hours (**Figure 3D**). Collectively, these findings highlight TDP1-EGF’s substantial therapeutic potential, suppressing excessive keratinocyte differentiation and scar formation while accelerating wound healing through enhanced scab detachment, stratum corneum regeneration, and neovascularization.

### 3.4 TDP1-EGF modulates keratinocyte proliferation in a murine model of epidermal barrier damage

**Figure 4:**
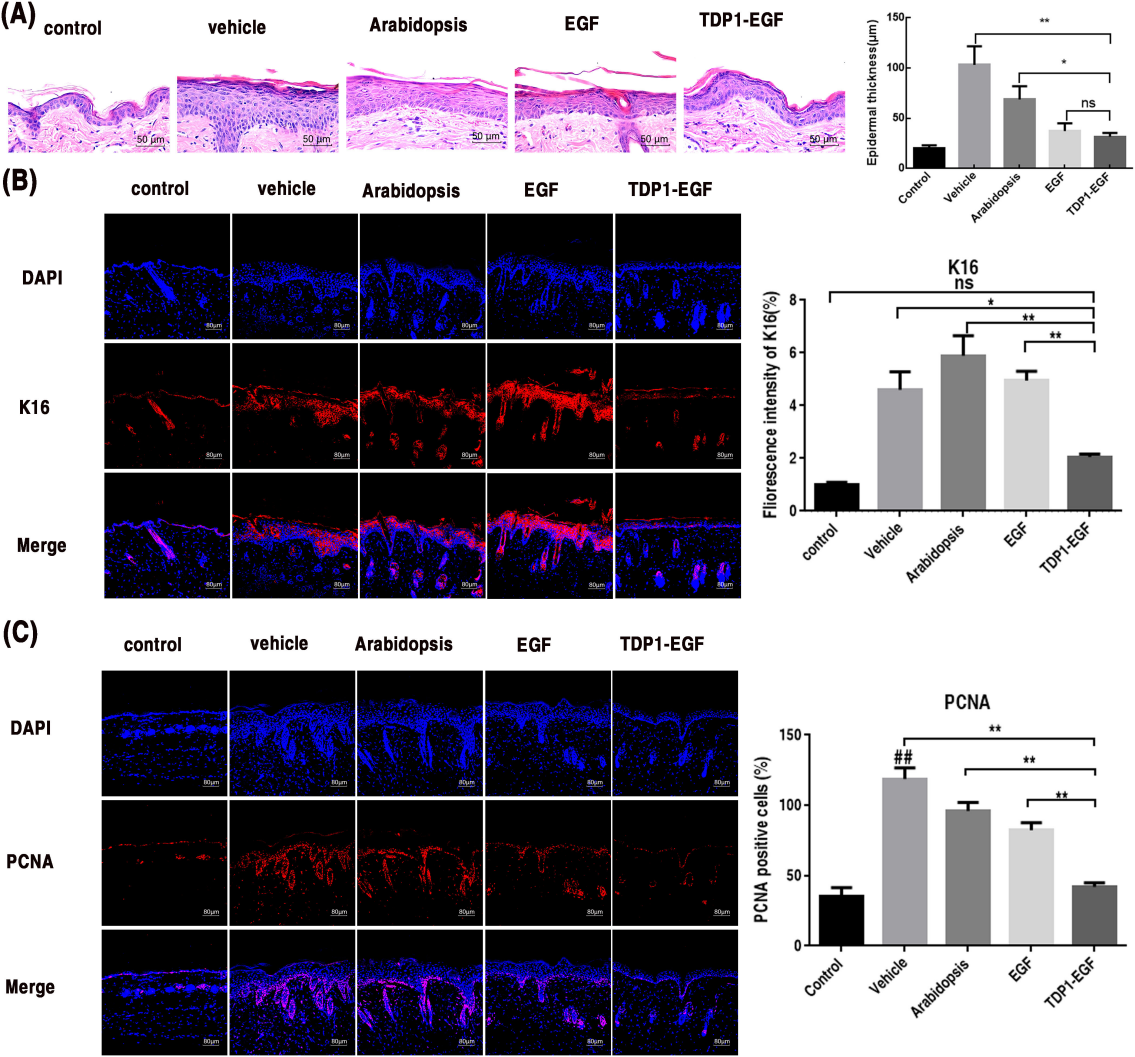
TDP1-EGF alleviates epidermal hyperplasia in mice. **(A)** Epidermal thickness alterations in dorsal skin assessed via hematoxylin and eosin (H&E) staining. **(B)** Keratin 16 (K16) expression levels in epidermal sections evaluated by immunofluorescence analysis. **(C)** Proliferating cell nuclear antigen (PCNA) expression levels in epidermal sections evaluated by immunofluorescence analysis. All graphical data are presented as mean ± SEM from four independent experiments. Statistical significance is denoted by ‘##’ (*P* < 0.01 vs. control), ‘*’ (*P* < 0.05 vs. vehicle), and ‘**’ (*P* < 0.01 vs. vehicle).

To evaluate the impact of TDP1-EGF on epidermal hyperplasia in mice, skin sections from each treatment group were examined using H&E staining and immunofluorescence. H&E staining revealed no significant differences in epidermal thickness between the TDP1-EGF and EGF groups or between the vehicle and *Arabidopsis* groups. However, the TDP1-EGF group displayed significantly thinner epidermis compared to the vehicle and *Arabidopsis* groups (**Figure 4A**). To further determine whether TDP1-EGF suppresses aberrant epidermal proliferation, immunofluorescence analysis was conducted to evaluate the expression of proliferation markers K16 and PCNA. The TDP1-EGF-treated group exhibited markedly reduced K16 and PCNA expression levels compared to the vehicle, *Arabidopsis*, and EGF groups. Strikingly, TDP1-EGF exerted a more potent inhibition of K16 and PCNA expression than EGF alone, with statistically significant differences. These results suggest that, while TDP1-EGF and EGF share similar biological functions in alleviating epidermal hyperplasia, TDP1-EGF’s enhanced transdermal efficacy—attributable to TDP1’s properties—confers a more pronounced regulatory effect on proliferation marker expression (**Figures 4B, C**).

### 3.5 Effects of TDP1-EGF on cell differentiation and lipid synthesis

**Figure 5:**
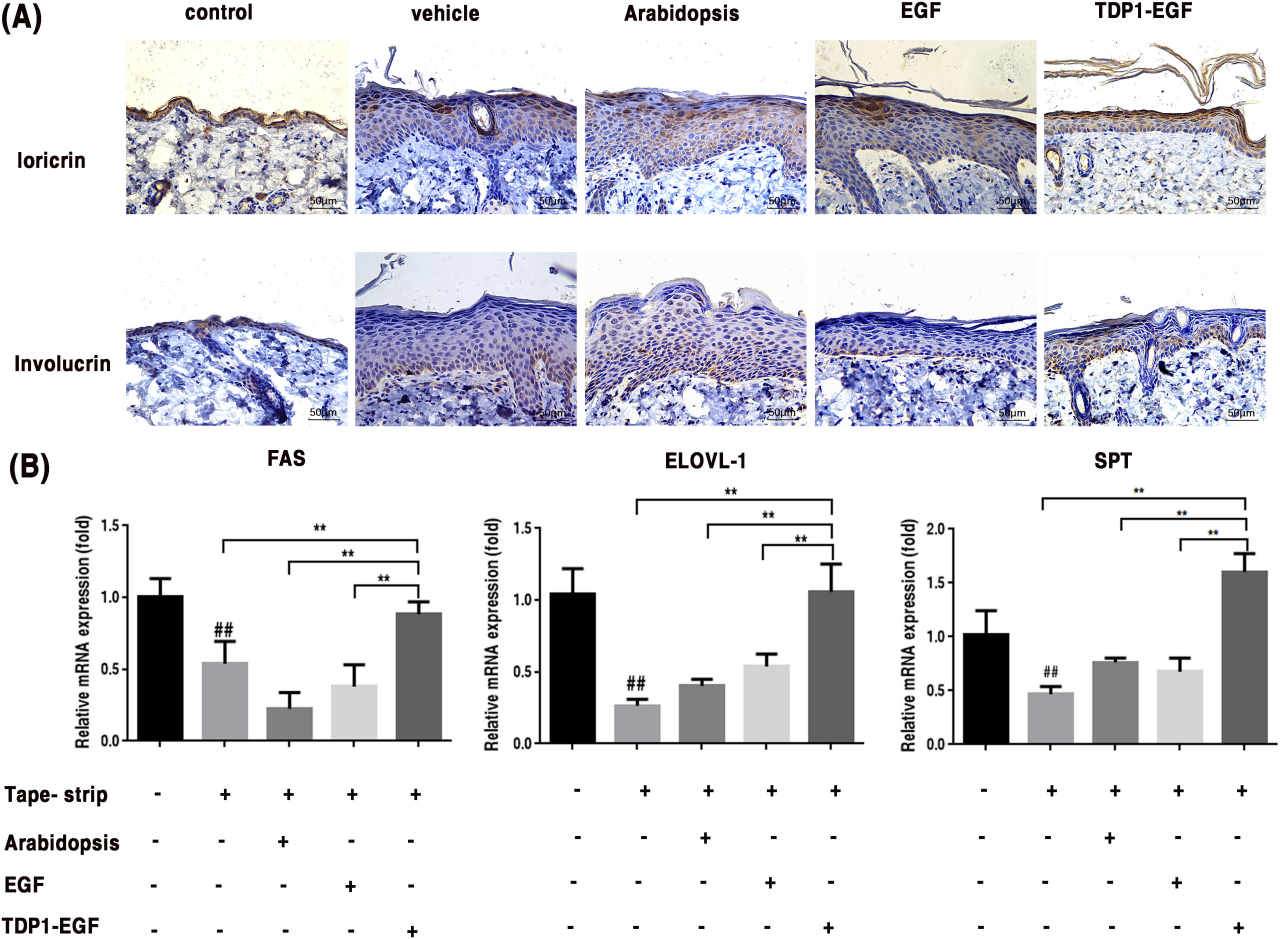
TDP1-EGF upregulates differentiation proteins and lipid synthesis genes to enhance skin barrier repair. **(A)** Expression levels of differentiation proteins involucrin and loricrin assessed via immunohistochemical analysis. **(B)** Transcript levels of lipid synthesis genes (*FAS*, *ELOVL-1*, and *SPT*) quantified by RT-qPCR. All graphical data are presented as mean ± SEM from four independent experiments. Statistical significance is denoted by ‘##’ (*P* < 0.01 vs. control), ‘*’ (*P* < 0.05 vs. vehicle), and ‘**’ (*P* < 0.01 vs. vehicle).

To elucidate TDP1-EGF’s role in epidermal barrier recovery, its effects on cell differentiation and lipid synthesis were assessed. Immunohistochemical analysis revealed that markedly elevated loricrin expression in the TDP1-EGF-treated group compared to the vehicle, *Arabidopsis*, and EGF groups, alongside a significantly thinner epidermis. Notably, loricrin in the TDP1-EGF group displayed a highly organized arrangement, in contrast to the reduced and disordered expression observed in the vehicle, *Arabidopsis*, and EGF groups (**Figure 5A**). Elongation of very long-chain fatty acids 1 (ELOVL-1), a key enzyme in the fatty acid elongation pathway, is essential for synthesizing ultra-long-chain fatty acids that serve as critical precursors for ceramide production. Serine Palmitoyltransferase (SPT), the rate-limiting enzyme in sphingolipid biosynthesis, catalyzes the initial step of sphingolipid synthesis, indirectly influencing ceramide generation. Fatty acid synthase (FAS), a multifunctional enzyme complex, drives the *de novo* fatty acids synthesis, providing fundamental components for ceramides, cholesterol esters, and other complex lipids. Collectively, these enzymes form an integrated network underpinning the synthesis of lipids vital for epidermal barrier integrity and function. Real-time quantitative PCR (RT-qPCR) analysis further demonstrated significantly elevated transcript levels of *SPT*, *FAS*, and *ELOVL-1* in skin tissue from the TDP1-EGF compared to the vehicle, *Arabidopsis*, and EGF groups, which showed no notable intergroup differences. Strikingly, TDP1-EGF surpassed EGF alone in upregulating lipid synthesis gene expression (**Figure 5B**). Primers sequences for RT-qPCR are provided in **Supplementary Table S2**. These results suggest that TDP1-EGF substantially enhances skin repair and regeneration by upregulating proteins involved in cell differentiation and lipid synthesis, an effect likely attributable to TPD1’s enhancement of EGF’s transdermal delivery within the fusion protein.

### 3.6 TDP1-EGF alleviates epidermal inflammation

**Figure 6:**
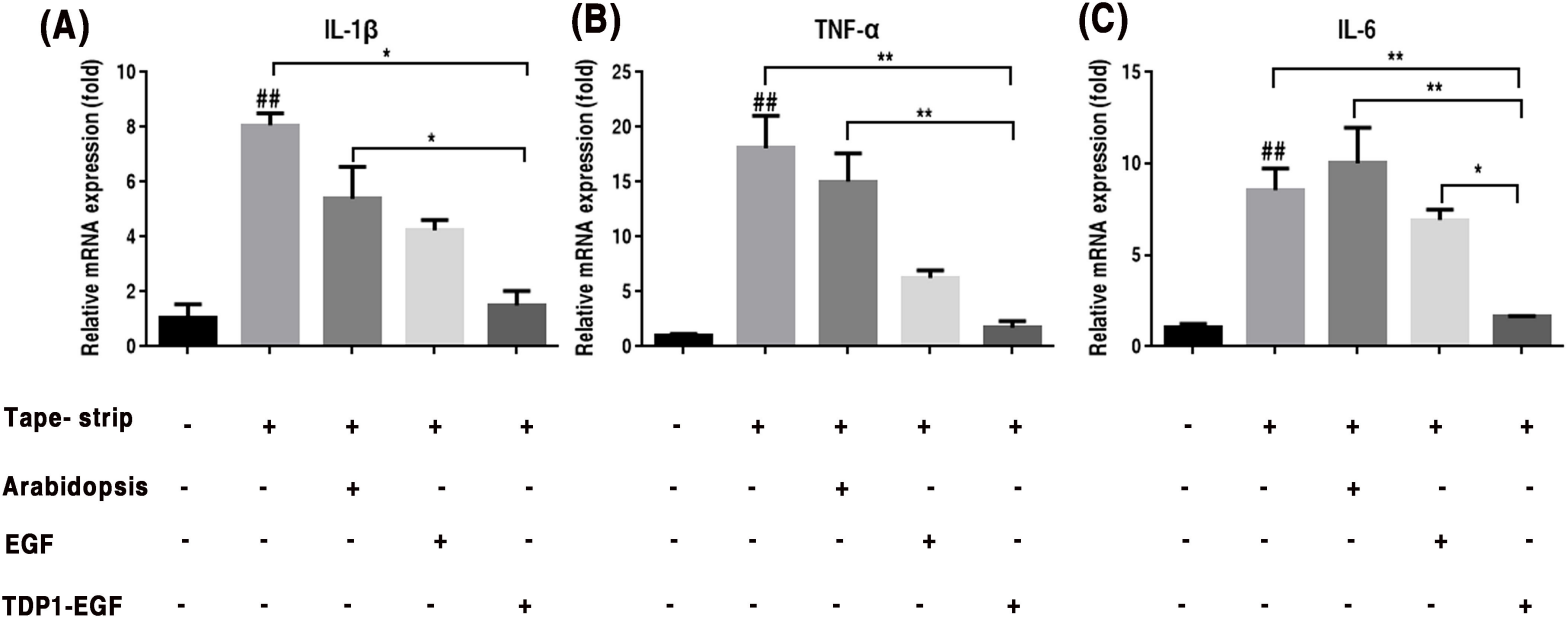
TDP1-EGF modulates inflammatory cytokine gene expression assessed by RT-qPCR. **(A)** To investigate the effect of TDP1-EGF on the expression levels of the inflammatory factor IL-1β. **(B)** To examine the effect of TDP1-EGF on the expression levels of the inflammatory factor TNF-α. **(C)** To assess the effect of TDP1-EGF on the expression levels of the inflammatory factor IL-6. Graphical data are presented as mean ±SEM from four independent experiments. Statistical significance is denoted by ‘##’ (*P* < 0.01 vs. control), ‘*’ (*P* < 0.05 vs. vehicle), and ‘**’ (*P* < 0.01 vs. vehicle).

To assess the impact of TDP1-EGF on the inflammatory response in epidermal barrier damage, mRNA expression of key pro-inflammatory cytokines (IL-1β, TNF-α, and IL-6) were quantified in skin tissue. Primers sequences for RT-qPCR are provided in **Supplementary Table S2**. RT-qPCR analysis demonstrated that the TDP1-EGF-treated group exhibited significantly reduced transcript levels of *IL-1β* and *TNF-α* compared to the vehicle and *Arabidopsis* groups, though no notable difference was observed between the TDP1-EGF and EGF groups (**Figures 6A, B**). Conversely, while *IL-6* expression showed no significant variation among the vehicle, *Arabidopsis* and EGF groups, the TDP1-EGF group displayed a pronounced downregulation of *IL-6* levels compared to all other experimental groups (**Figure 6C**). These results indicate that TDP1-EGF markedly suppresses the expression of inflammatory cytokines *IL-1β*, *TNF-α* and *IL-6*, exerting a more potent anti-inflammatory effect than EGF alone. This suggests that TDP1-EGF not only enhances epidermal barrier repair but also effectively modulates the inflammatory response associated with barrier damage.

## 4 Discussion

Epidermic growth factor is a protein known to promote wound closure and epidermal regeneration (Li et al., 2021). However, the effectiveness of topical application of EGF at the wound site can be hampered by the poor transdermal delivery of the protein into the skin tissue, primarily because of the size of EGF, which is greater than 6,000 Da, making it difficult to penetrate the corneal layer, which only allows molecules of less than 500 Da to readily pass through (Bos and Meinardi, 2000; Adamson and Rees, 1981). One approach to improve EGF’s skin penetration is to fuse it with a highly efficient transdermal peptide. Given that this strategy entails the construction and expression of a recombinant protein, the expression system employed must prioritize product safety. While prokaryotic expression systems are well-established and widely utilized for exogenous protein production. Unfortunately, expressing exogenous proteins in a prokaryotic expression system such as the *E. coli* expression system may not be desirable if the expressed proteins are for human or animal usage since there is a risk of contamination by endotoxins, which pose a risk to health (Wakelin et al., 2006). Additionally, *E. coli*-based systems demand costly fermenters, stringent conditions, and offer limited scalability (Chaudhary et al., 2024). Thus, expressing exogenous proteins in a plant expression system would be more desirable (Chen et al., 2023; Huang and McDonald, 2012; Moustafa et al., 2016).

Currently, numerous plants are capable of enhancing the expression levels of foreign proteins through transient expression, thereby meeting commercial standards (Ruhlman et al., 2007). For stable nuclear transformation, optimizing expression vectors is widely acknowledged as an effective approach to enhance recombinant protein yields. For example, Ruggiero et al. (2000) demonstrated that incorporating an enhanced 35S promoter and 35S terminator in tobacco enabled the production of approximately 3 g of purified recombinant collagen per 100 kg of powdered plant material (Ruggiero et al., 2000). Similarly, Suo et al. (2006) reported substantially improved expression levels of bone morphogenetic protein 2 (BMP-2) in tobacco by employing the CaMV 35S promoter and codon optimization strategies (Suo et al., 2006). In our prior study, despite employing comparable optimization strategies, the expression level of the target protein was limited to 0.016% of total soluble protein, falling below commercial standard (**Figure 1D**). To achieve commercialization requirements, additional optimization approaches will be pursued, including optimizing *Arabidopsis thaliana* growth conditions, improving the post-translational stability of TDP1-EGF, and exploring co-expression with molecular cofactors. In a related study, Wirth et al. (2004) expressed hEGF in tobacco, targeting its accumulation to either the cytoplasm or the apoplast. Cytoplasmic accumulation resulted in a minimal expression level of 0.00001% of total soluble protein, whereas apoplast-targeted accumulation significantly elevated hEGF expression to 0.11% (Wirth et al., 2004). Similarly, Qiang et al. (2020) enhanced the stability and expression levels of a fusion protein by expressing oleosin-EGF in *Arabidopsis thaliana* seeds, with the highest expression level reaching 14.83 ng/μL (Qiang et al., 2020). These investigations offer valuable insights and approaches for further optimizing recombinant protein expression in plant-based systems.

Our data demonstrate that TDP1-EGF is expressed across all *A. thaliana* tissues, including stems, roots, leaves, and flowers, as confirmed by RT-qPCR analysis, suggesting that *A. thaliana* serve as an effective bioreactor for the commercial production of TDP1-EGF (**Figure 1B**). Consequently, *A. thaliana* represents a promising plant-based bioreactor system for commercial applications. The transdermal peptide TDP1 can be linked to EGF via fusion expression or chemical coupling. Notably, EGF activity and TDP1 transdermal properties remained unaffected when expressed as the TDP1-EGF fusion protein (**Supplementary Figure S4**). Our findings revealed that the TDP1-EGF fusion protein reached peak accumulation during the second week of *A. thaliana* growth. Additionally, the fusion protein exhibited no impact on the phenotype or genetic patterns of *A. thaliana*, supporting the feasibility of fusing the transdermal peptide TPD1 with EGF. In experiments accessing epidermal barrier repair, hydrogel supplemented with total protein extracted from wild-type *A. thaliana* demonstrated superior repair promotion compared to hydrogel alone, suggesting that plant-derived compounds may enhance repair processes and potentially synergize with TDP1-EGF to improve epidermal barrier recovery. Taking into account both safety and efficacy, a wealth of current data has indicated that the optimal concentration of EGF is 1 μg per 30g (Fabi and Sundaram, 2014; Esquirol Caussa and Herrero Vila, 2015). Accordingly, the TDP1-EGF hydrogel was formulated with a protein-to-hydrogel ratio of 1μg per 30 g to attain the optimal concentration. After expression in *A. thaliana*, TDP1-EGF was extracted as the soluble protein fraction, incorporated into a hydrogel, and applied directly to the damaged skin. Its efficacy in skin repair was then compared with that of hydrogel alone and hydrogel containing standard EGF. The result demonstrated significantly accelerated recovery of the damaged skin with the TDP1-EGF hydrogel, indicating that fusing EGF with TDP1 substantially enhanced EGF’s efficacy in skin repair. This improvement stemmed not from superior intrinsic activity of the fusion protein over EGF alone, but rather from TPD1-mediated enhancement of EGF’s transdermal delivery (**Figure 3A**). The safety and efficacy of biological approaches to transdermal drug delivery have been consistently substantiated. For example, Rothbard et al. (2000) reported that the conjugation of Cyclosporine A with an arginine oligomer effectively penetrated the epidermal barrier and exerted anti-inflammatory effects (Rothbard et al., 2000). Similarly, Kim et al. (2006) successfully transduced Botulinum Neurotoxin (BoNT), an anti-aging compound, into mouse skin using the cell-penetrating peptide Pep-1 (Kim et al., 2006). To further assess the efficacy of transdermal peptides, multiple parameters were employed to evaluate TDP1-EGF ‘s effectiveness, and the collective dare underscore the benefits of fusing EGF with TDP1, positioning this approach as a promising enhancement for EGF application in treating damaged skin.

Our prior research has demonstrated that a healthy epidermal barrier is formed by the proliferation and differentiation of keratinocytes (Wikramanayake et al., 2014). Damage to the skin barrier leads to abnormal proliferation of keratinocytes, which in turn causes continuous deterioration of the barrier (Yang et al., 2020; Patrick et al., 2021). The key to repairing the barrier is to normalize the differentiation of abnormally proliferating keratinocytes. An important measure of epidermal barrier function is the TEWL value (Montero-Vilchez et al., 2022; Lodén et al., 1999). A growing body of research has established that EGF effectively promotes skin wound healing and epidermal regeneration, primarily by regulating proteins associated with cell proliferation, differentiation, and migration (Gibbs et al., 2000). Our results demonstrated that topical application of TDP1-EGF effectively promoted the recovery of epidermal barrier damage in the dorsal skin of mice, outperforming EGF alone, as evidenced by increased skin moisture levels observed via dermoscopy (**Figures 3B, C**). These findings confirmed our hypothesis that transdermal peptides, such as TPD1, facilitate the delivery of EGF to keratinocytes in the deeper epidermal layers, enabling precise exertion of its therapeutic effects. H&E staining revealed that, following tape stripping, the epidermis thickened due to injury; however, no significant difference in epidermal thickness was observed between the TDP1-EGF-treated group and the negative control group (**Figure 4A**). Collectively, these results indicate that TDP1-EGF treatment prevents aberrant keratinocyte proliferation.

EGF regulates differentiation, migration, proliferation, and other biological activities of keratinocytes through a mitogen-activated protein kinase (MAPK)-dependent mechanism and PI3K/AKT signal transduction (Haase et al., 2003; Chen et al., 2022a). TDP1-EGF was shown to modulate the expression of key differentiation markers, including involucrin and loricrin, thereby promoting orderly differentiation of stratum corneum cells to maintain skin barrier integrity (**Figure 5A**). Loricrin and involucrin are pivotal proteins in the differentiation of epidermal keratinocytes (Lee et al., 2014). Involucrin expression signals the onset of terminal differentiation in keratinocytes, with elevated levels closely linked to cellular flattening and keratinization (LaPres and Hudson, 1996). Research has demonstrated that the promoter region of the involucrin gene contains transcription factor binding sites that can be activated by the MAPK pathway, enabling EGF to enhance involucrin gene transcription by activating this pathway (Ghahary et al., 2001; Gibbs et al., 2000). Additionally, researchers have confirmed that EGF can upregulate involucrin expression in the A431 cell line and promote its differentiation process (Rosdy, 1988). To date, no direct evidence supports EGF-mediated regulation of loricrin expression. However, our prior research demonstrated that theTDP1-EGF fusion protein modulates the expression of differentiation proteins, including involucrin and loricrin, thus facilitating terminal keratinocyte differentiation. One plausible explanation for this finding is that EGF modulates the expression of differentiation proteins via well-established signaling pathways, including PI3K/AKT and MAPK. Ceramides and free fatty acids are critical components in sustaining normal epidermal barrier function. When the epidermal barrier is compromised, the synthesis of cholesterol, free fatty acids, and ceramides increases to facilitate the repair process of the epidermal barrier (Rosdy, 1988). Several studies have demonstrated that the expression of serine palmitoyltransferase (SPT) is significantly up-regulated during vascular repair, promoting ceramide synthesis and thereby accelerating wound healing (Uhlinger et al., 2001). Additionally, ElovL-1 and fatty acid synthase (FAS) are pivotal in sustaining epidermal barrier integrity. ElovL-1, in particular, is recognized as a critical lipid synthetase indispensable for epidermal barrier function. Notably, mice deficient in ElovL-1 exhibit severe epidermal barrier defects and typically die shortly after birth (Sassa et al., 2013). Furthermore, ω-hydroxy-ceramide (ω-OH-Cer), anchored to the outer surface of the keratinocyte lipid envelope (CLE) and associated with involucrin, constitutes a vital lipid component of the stratum corneum (SC). The biosynthesis of ω-OH-Cer is closely linked to FAS activity, highlighting the interdependence of these pathways in maintaining epidermal barrier function (Ge et al., 2023). TDP1-EGF was found to upregulate the expression of lipid synthesis genes, including *ELOVLs, FAS*, and *SPT*, as evidenced by **Figure 5B**. Given that stratum corneum cell proliferation, differentiation, and migration are closely related to EGFR expression and lipid composition, this upregulation suggests that TDP1-EGF may initiate epidermal repair signaling by binding to EGFR. Future studies will further elucidate the mechanisms underlying TDP1-EGF’s effects.

In addition to enhancing cell proliferation, differentiation, and migration, TDP1-EGF also mitigated inflammation resulting from damage to skin tissue. It has been reported that the abnormal proliferation of structural proteins and the abnormal expression of inflammatory factors are two important factors in the dysplasia of stratum corneum cells (Evtushenko et al., 2021; Jiao et al., 2022). PCNA and K16 are markers of abnormal cell proliferation, and these two markers can be used to estimate the extent of malignant lesions via histological analysis (Krecicki et al., 1999; Paladini and Coulombe, 1998). In this study, TDP1-EGF was found to downregulated the expression of PCNA and K16 proteins (**Figure 4B**). To date, no studies have demonstrated that EGF alone inhibits the expression of PCNA and K16. From another perspective, inflammatory factors, such as TNF-α and IL-1β can activate multiple intracellular signaling pathways, thereby promoting the expression of PCNA (Krecicki et al., 1999; Paladini and Coulombe, 1998). For instance, TNF-α has been shown to enhance PCNA gene transcription and expression by activating the NF-κB signaling pathway, which facilitates the nuclear translocation of NF-κB and its binding to the promoter region of the PCNA gene (Ji et al., 2016; De Simone et al., 2015; Niu et al., 2022). Furthermore, studies have indicated that inflammatory factors can induce a stress response in cells. In response to such stress, cells in the psoriatic epidermis may enhance the nuclear translocation of Nrf2. Once in the nucleus, Nrf2 can bind to the promoter region of the K16 gene, activate its transcription, and increase the expression of stress-related proteins such as K16 (Yang et al., 2017). Therefore, we hypothesize that the TDP1-EGF fusion protein may indirectly suppress the expression of proliferative proteins, such as PCNA and K16, by attenuating inflammatory cytokine levels in specific contexts, thereby ameliorating dysplasia in damaged regions of the murine epidermal barrier. Further validation confirmed that TDP1-EGF modulates inflammatory cytokine expression, with study results demonstrating significant downregulation of genes encoding IL-1β, TNF-α, and IL-6 in skin tissue following TDP1-EGF application, supporting its role in reducing inflammation (**Figures 6A–C**). This anti-inflammatory effect likely enhances TDP1-EGF’s efficacy in repairing damaged epidermal barriers by mitigating inflammation-induced exacerbation of injury.

Although the development of genetically modified (GM) plants expressing skincare products holds considerable promise, several critical challenges impede its advancement. First, plant bioreactor technology lags behind prokaryotic expression systems in sophistication, resulting in lower protein yields and reduced market competitiveness (Law et al., 2025). Second, global regulatory frameworks governing the expression of pharmaceutical proteins in plants remain underdeveloped, lacking standardized guidelines (Turnbull et al., 2021). Regulatory policies differ markedly across countries, posing compliance challenges for companies. Moreover, therapeutic proteins derived from GM plants must satisfy stringent safety, efficacy, and quality control standards to qualify for clinical trials, a milestone achieved by only a handful of such proteins to date (Burnett and Burnett, 2020). Nevertheless, advancements in plant biotechnology, alongside ongoing refinements in regulatory frameworks and the intrinsic benefits of transgenic plants—including enhanced safety, cost-effective production, scalability, and broad applicability across diverse sectors—position plant-based expression systems as an emerging cornerstone for skincare product manufacturing.

## 5 Conclusion

This study demonstrates the feasibility of expressing the TDP1-EGF fusion protein in *Arabidopsis thaliana*, achieving a peak expression level of 142.05 ng/mL, which constitutes 0.016% of total soluble protein. Our results reveal that the transdermal absorption rate of TDP1-EGF fusion protein surpasses that of EGF alone. Significantly, this research provides the first evidence that *A. thaliana*-expressed TDP1-EGF fusion protein effectively ameliorates physically induced epidermal barrier damage through multiple mechanisms: (1) suppressing inflammation, (2) alleviating epidermal hyperplasia, (3) upregulating lipid synthesis, (4) enhancing keratinocyte differentiation, and (5) accelerating epidermal barrier repair. These findings lay a solid theoretical and practical foundation for leveraging *A. thaliana*-expressed TDP1-EGF fusion protein in medical and skincare applications, particularly for epidermal barrier repair. Moreover, this study offers valuable insights into the potential of plant-based bioreactors systems for advancing skin health solutions.

## Data Availability

All data supporting the findings of this study are available within the paper and its **Supplementary Information**.
