# Supplementary material for: Expression of an epidermal growth factor-transdermal peptide fusion protein in Arabidopsis thaliana and its therapeutic effects on skin barrier repair
Source: Front Plant Sci. 2025 Apr 4;16:1573193. doi: 10.3389/fpls.2025.1573193 (PMC12007040; doi:10.3389/fpls.2025.1573193)
Supplement: Supplementary file 1 [file DataSheet1.pdf]

Supplementary Material

1 Supplementary Figures

Figure 1

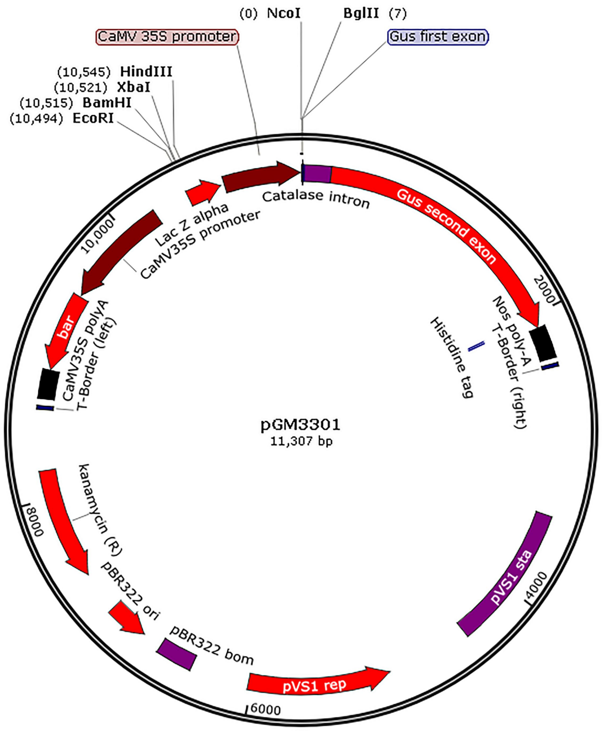

Supplementary Figure 1.(A) Map of plant expression vector pGM3301; The GUS region is replaced by the target genes TDP1-EGF by restriction endonucrenases *NcoI* and *BstEII*.

Figure 2

EGF sequencing results

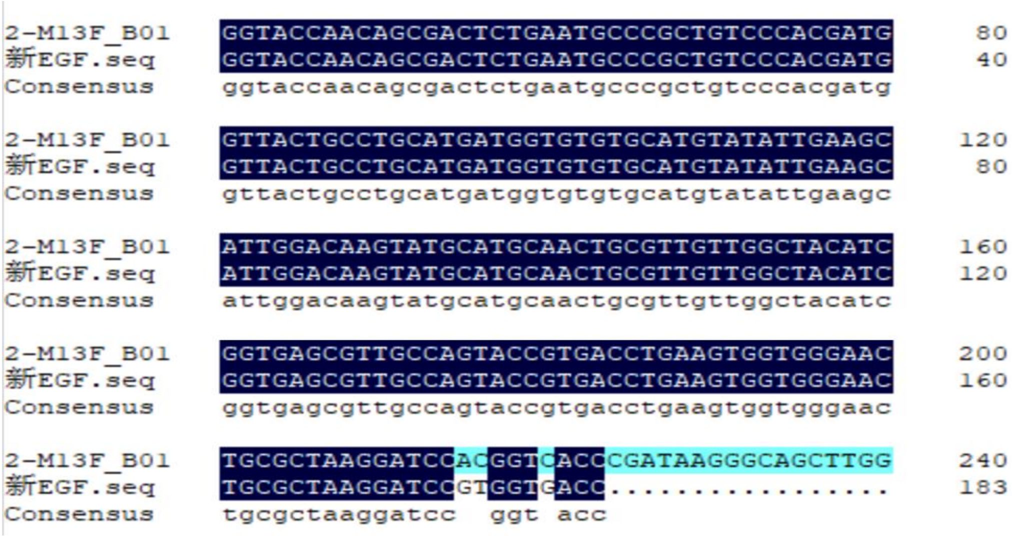

TDP1 sequencing results

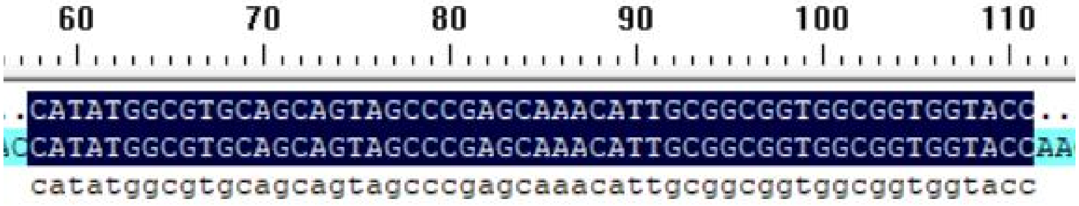

Supplementary Figure S2. (A) TDP1-EGF genetic sequence (239bp) (B) Sequencing results at the bio company (GENEWIZ,China), and the mutation of two bases does not affect the expression of the target protein.

Figure 3

(A)

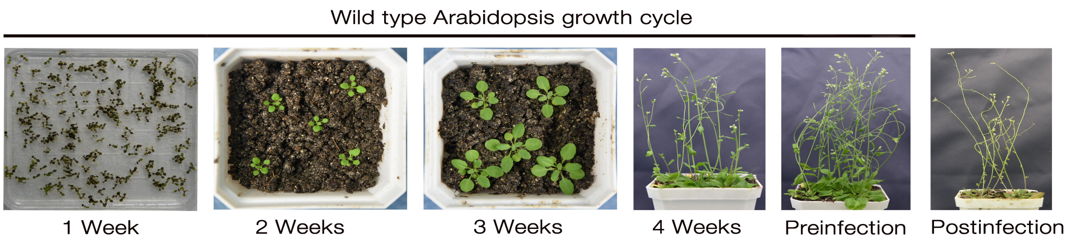

(B)

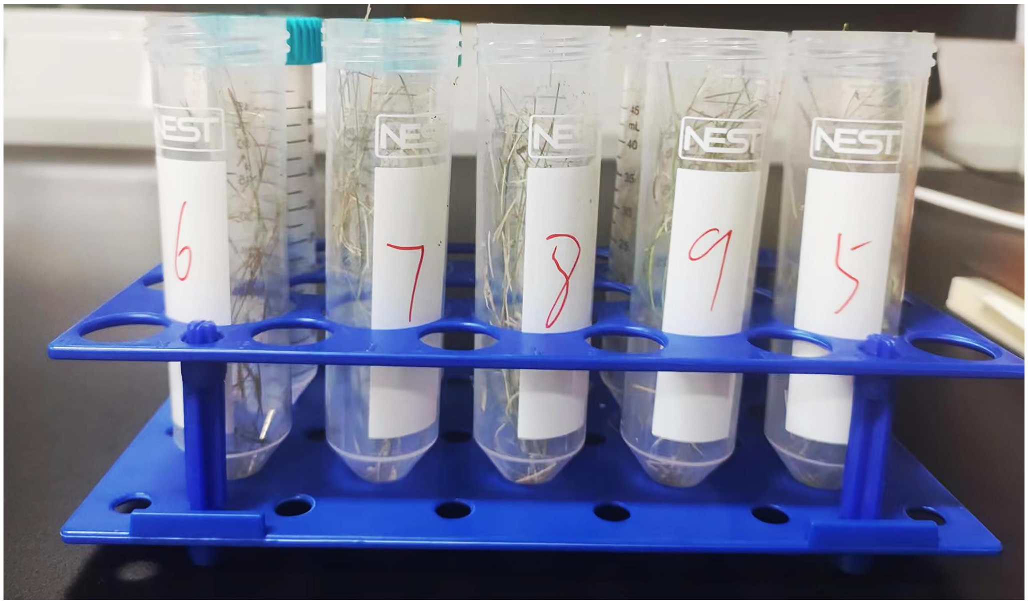

Supplementary Figure S3. (A) Wild type Arabidopsis growth cycle (B) The T2 seeds of transgenic Arabidopsis thaliana were harvested from a single plant

2 Supplementary Tables

TABLE 1

Restriction endonuclease	genetic sequence	source
NcoI	CCATGG	NEB, United States
KpnI	GGTACC	NEB, United States
BstEII	GGTGACC	NEB, United States

Supplementary TABLE 1. Construction of plant expression vector through the required enzyme restriction sites.

TABLE 2

gene	Host	Forward primer sequence(5' > 3')	Reverse primer sequenc (5' > 3')
SPT	Mouse	GTTGCAGGAGCGTTCTGATCT	GGCCGGACACGATGTTGTAG
ELOVL-1	Mouse	CCTGAAGCACTTCGGATGGT	GCTGGAGCTCCATTTTGCTG
FAS	Mouse	GGCCCCTCTGTTAATTGGCT	CGCTTGTTGGTGGACACTTG
IL-1β	Mouse	TGTGTAATGAAAGACGGCACACC	GTATTGCTTGGGATCCACACTCTC
TNF-α	Mouse	CAGGCGGTGCCTATGTCTCA	GGCTACAGGCTTGTCACTCGAA
IL-6	Mouse	TCCTCTCTGCAAGAGACTTCCATC	TGGTTGTCACCAGCATCAGTCC
GAPDH	Mouse	CGAAGGTGGAAGAGTGGGAGTTG	AGAAGGTGGTGAAGCAGGCATC

Action	Arabidopsis	GGTAACATTGTGCTCAGTGGTGG	AACGACCTTAATCTTCATGCTGC
TDP1-EGF	Human	AACAGCGACTCTGAATGCCCGC	GCGCAGTTCCCACCACTTCAGGTC

Supplementary TABLE 2. Primer gene sequences required for qPCR detection.
