# Supplementary material for: Expression of an epidermal growth factor-transdermal peptide fusion protein in Arabidopsis thaliana and its therapeutic effects on skin barrier repair
Source: Front Plant Sci. 2025 Apr 4;16:1573193. doi: 10.3389/fpls.2025.1573193 (PMC12007040; doi:10.3389/fpls.2025.1573193)
Supplement: Supplementary file 2 [file DataSheet2.docx]

Supplementary Material

# Supplementary Figures


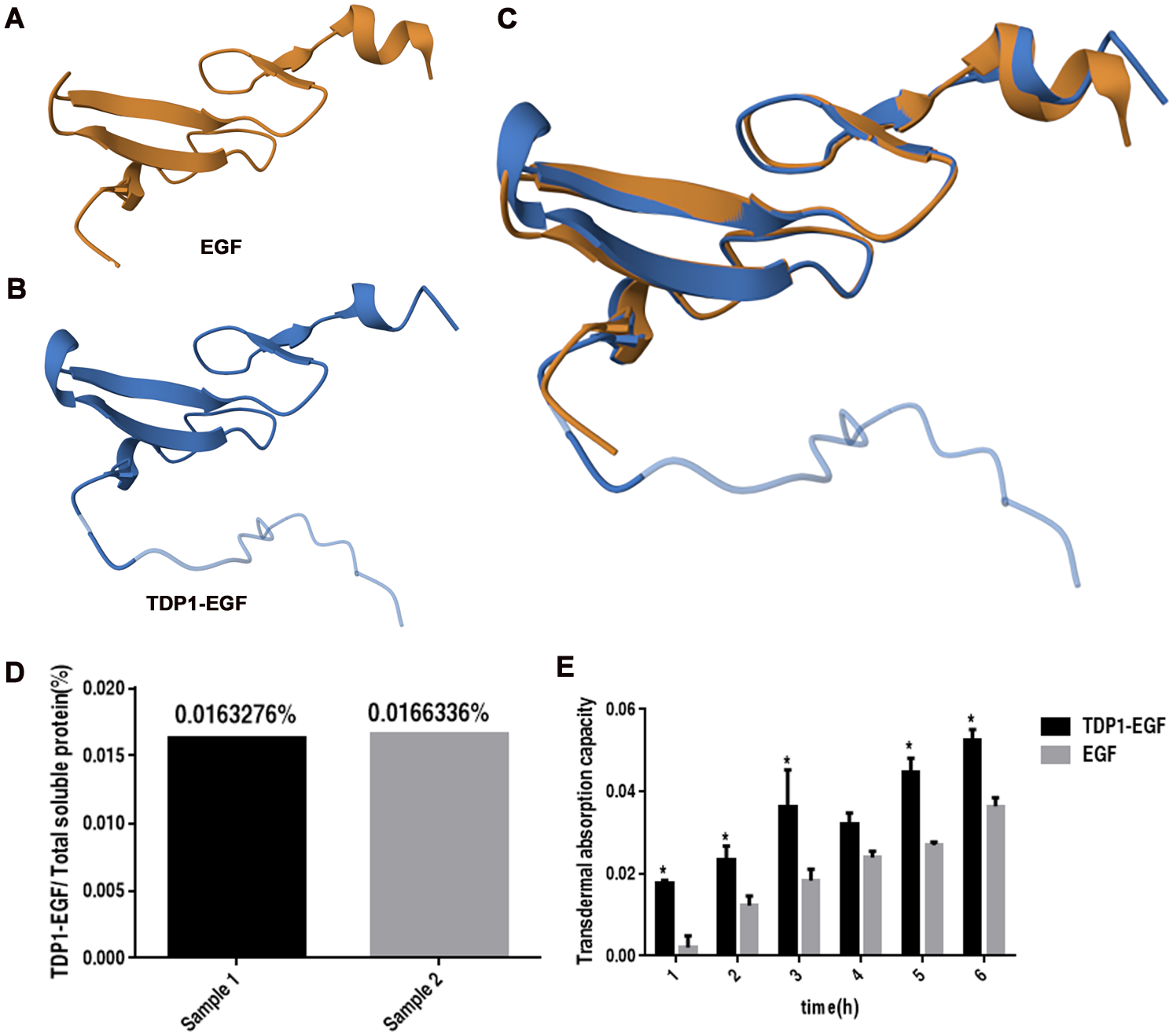


**Supplementary Figure 4.** (A) Molecular structure prediction of EGF. (B) Molecular structure prediction of fusion protein TDP1-EGF. (C) Comparison of molecular structure prediction of fusion proteins TDP1-EGF and EGF
